# New Approaches to Thyroid Hormones and Purinergic Signaling

**DOI:** 10.1155/2013/434727

**Published:** 2013-07-17

**Authors:** Gabriel Fernandes Silveira, Andréia Buffon, Alessandra Nejar Bruno

**Affiliations:** ^1^Instituto Federal de Educação, Ciência e Tecnologia do Rio Grande do Sul, Porto Alegre (IFRS-POA), Rua Ramiro Barcelos 2777, 90.035-007 Porto Alegre, RS, Brazil; ^2^Departamento de Análises Clínicas, Faculdade de Farmácia da Universidade Federal do Rio Grande do Sul (UFRGS), Avenida Ipiranga 2752, 90610-000 Porto Alegre, RS, Brazil

## Abstract

It is known that thyroid hormones influence a wide variety of events at the molecular, cellular, and functional levels. Thyroid hormones (TH) play pivotal roles in growth, cell proliferation, differentiation, apoptosis, development, and metabolic homeostasis via thyroid hormone receptors (TRs) by controlling the expression of TR target genes. Most of these effects result in pathological and physiological events and are already well described in the literature. Even so, many recent studies have been devoted to bringing new information on problems in controlling the synthesis and release of these hormones and to elucidating mechanisms of the action of these hormones unconventionally. The purinergic system was recently linked to thyroid diseases, including enzymes, receptors, and enzyme products related to neurotransmitter release, nociception, behavior, and other vascular systems. Thus, throughout this text we intend to relate the relationship between the TH in physiological and pathological situations with the purinergic signaling.

## 1. An Overview about Thyroid Hormones

Thyroxine (T4), triiodothyronine (T3), and reverse triiodothyronine (rT3) are the three main forms of thyroid hormones (TH) found in the bloodstream. T4 and T3 are essential for normal functioning of different systems. TH play a central role in the growth, proliferation, differentiation, apoptosis, development, neurotransmission, behavior, and metabolic homeostasis [1–3]. 

The most common effects of TH are those on metabolism. TH affect the intermediary metabolism of proteins, lipids, and carbohydrates in almost all tissues. T3 target genes were studied by microarray assay in cell lines of hepatocellular carcinoma and detoxification; cell adhesion, signal transduction, cell migration, transcription factors and cell cycle oncogenesis were recognized to be regulated by treatment with T3 [4].

T3 is also able to induce vasodilatation through mechanisms not yet fully elucidated [5]. The positive inotropic, dromotropic, and chronotropic heart rate are also increased by TH [6]. These effects are associated with an increase in sensitivity of adrenergic and cardiac receptors, as well as, an increased synthesis of myosin [7, 8]. Thus, hyperthyroidism is common to observe responses such as tachycardia and cardiac hypertrophy [6].

In the nervous system, the TH act mainly on the mechanisms involved in the central and peripheral neurotransmission by increasing the synthesis and sensitivity to catecholamines [9]. Studies have shown a reduction in the release of glutamate and NMDA receptor expression in rat brain following the induction of hypothyroidism surgery [10, 11]. The precise mechanism by which the hypothyroidism induces neurological problems such as memory impairment is not yet fully elucidated. However, cognitive disturbances described in patients with untreated hypothyroidism are often associated with decreased excitability in the central nervous system (CNS) [12].

In the early stages of brain development in mammals, TH promote cell proliferation and subsequently act by inhibiting proliferation and stimulating cell differentiation [13]. The lack of TH during the period of neurogenesis (up to six months of postnatal life) results in irreversible neurological deficits and is accompanied by multiple morphological brain alterations [14]. TH deficiency during the fetal and neonatal periods results in disturbances in the process of neuronal migration, reduction of synaptogenesis, defective myelination, and the synthesis of neurotransmitters [13–15].

Although the secretion of T4 from thyroid is several times greater than T3, the later is roughly two to three times more effective than the former. 

Since 1970, most studies have mainly described the concept of genomic action of TH through the transcriptional regulation of genes responsive to these hormones [16, 17]. T3 binds to specific high affinity receptors called thyroid receptors (TRs) which belong to the super family of nuclear receptors and mediate multiple effects on the phenotype, proliferation, and gene expression of cultured normal mammary epithelial cells [18, 19]. TRs bind to DNA at specific sequences and the TR response element (TREs) in target gene promoters. 

In addition to nuclear effects mediated by these receptors, nongenomic actions of TH have been recently characterized [20, 21]. These activities include effects on the rapid plasma membrane [22] and cytoplasmic organelles [20]. Many of the rapid effects mediated by these hormones are not changed by the use of inhibitors of transcription and translation [21]. Moreover, it is widely demonstrated that membrane-initiated rapid responses to TH include alterations in ionic fluxes and in membrane potentials [23, 24]. Some authors also discuss the possible presence of isoforms of nuclear receptors on the plasma membrane [25]. It also appears that regulation of the actin cytoskeleton by T4 facilitates the interaction of transmembrane integrins with laminin, the principal extracellular matrix (ECM) protein in developing brain and a product of glial cells. This nongenomic action of TH on ECM could influence relationships of nerve cells during brain development [21]. TH are also able to modulate in vivo and in vitro the vimentin phosphorylation and expression in rat testis and cerebral cortex through genomic [26] and nongenomic mechanisms. In this context, the authors of [27] have demonstrated that T3 provoked vimentin hyperphosphorylation and association/aggregation into the cytoskeletal fraction of rat testicular cells. This effect was totally independent on protein synthesis, characterizing a nongenomic action for this hormone. Moreover, this effect was dependent on intra- and extracellular Ca2 levels, and vimentin hyperphosphorylation was prevented by appropriate blockers of Ca2 influx through L-VDCC (voltage-dependent Ca2+ channel). These findings suggest that T3 can play important nongenomic roles in the reorganization of the cytoskeleton, regulating cell physiology rat testis [26].

Disorders of the thyroid gland are among the most common endocrine maladies. Hyperthyroidism is characterized by nervousness, anxiety, physical hyperactivity, weight loss, increased perspiration, increase in metabolic routs, and, in the most severe situations, seizures. Hypothyroidism is the most prevalent form of thyroid disease and symptoms may include memory and learning impairment, depression, psychotic behavior, retarded locomotor ability, somnolence, progressive intellectual deterioration, and, in extreme cases, coma. 

Recently, TH and the diseases involved were related to the purinergic system. Our group has demonstrated the involvement of some key symptoms of thyroid dysfunction in the purinergic system in different tissues and biological samples, as it will be described throughout this review.

## 2. An Overview about Purinergic System

The signaling components in the purinergic system are ATP, P2 receptors, adenosine, adenosine receptors, ectonucleotidases, and nucleoside transporters. 

Nucleosides and nucleotides play their actions through activation of specific membrane purinoceptors. There are two purinergic receptor families named P1 and P2, which were identified in 1978 [28]. Purinergic receptors were subdivided on the basis of pharmacology (e.g., response to specific agonists) and molecular cloning [29, 30]. Adenosine is the endogenous agonist of P1 receptors family, which is composed by four subtypes of G-protein-coupled receptors named A1, A2A, A2B, and A3 [28]. The P2 receptor family is composed by P2X and P2Y receptors subtypes. The P2X receptors are ligand-gated cation channels composed of homo- or heterotrimeric P2X subunits, and the P2Y receptors are seven transmembrane G-protein-coupled receptors [28]. There are seven known P2X receptor subtypes (P2X1–7) and eight P2Y receptor subtypes (P2Y1, P2Y2, P2Y4, P2Y6, P2Y11, P2Y12, P2Y13, and P2Y14) [31].

Adenosine is involved in various physiological and pathological processes, both in the periphery and in the CNS [32, 33]. 

The inhibitory actions of adenosine on neurotransmitter release are mediated by specific plasma membrane A1 receptors [34, 35]. The A1 adenosine receptor is the most prevalent adenosine receptor subtype, having both a high level of expression and a widespread distribution [33]. Actions due to adenosine A1 receptors activation have been proposed to result from coupling to inhibitory protein Gi, inhibition of adenylate cyclase, inhibition of presynaptic voltage-sensitive Ca2+ channels, and activation of postsynaptic K+ channels [36].

Furthermore, adenosine plays a significant role in intracellular signal transduction mechanisms depressing the release of thyroid-stimulating hormone (TSH) by A1 receptor activation [37, 38].

The levels of extracellular adenosine are increased during periods of high metabolic demand, such as seizures, ischemia, hypoxia, and stressful challenges [39, 40].

In addition, adenosine-mediated inhibitory influences on nociceptive reflex responses have been demonstrated in experimental and clinical situations [41, 42]. It is known that the antinociceptive properties of adenosine are associated with adenosine A1 receptor activation at spinal sites [41].

Studies performed in mice lacking the adenosine A1 receptor confirm the involvement of adenosine in motor activity, exploratory behavior, anxiety, and aggressiveness [43]. Furthermore, adenosine antagonists like caffeine promote wakefulness, aggressive behavior in rats, and nervousness and irritability in man [44], while adenosine analogues counteract these effects [45].

Adenosine is also able to attenuate the deleterious consequences of reactive oxygen species (ROS) through A1 receptors activation in rat hippocampal slices [46]. In addition, oxidative stress induces expression of the A1 receptors [47], which provide cytoprotection in the CNS [48].

ATP is an excitatory synaptic transmitter in both central and peripheral nervous systems [30, 49]. Extracellular ATP, at micromolar concentrations, induces significant functional changes in a wide variety of cells and tissues. It has been shown that ATP is involved in the interaction between neurons and glial cells, homeostasis [50], and inflammatory reactions in the brain [50]. In cultured astrocytes, ATP induces differentiation, proliferation, and reactive astrogliosis in tissue injury [51]. ATP can be released from the cytosol of damaged cells or from exocytotic vesicles and/or granules contained in many types of secretory cells. Furthermore, the cellular ATP level is an important determinant for cell death. ATP signaling can trigger excitotoxicity via activation of calcium-permeable P2X7 purinergic receptors. Sustained activation of P2X7 receptors in vivo causes lesions that are reminiscent of the major features of demyelination, oligodendrocyte death, and axonal damage [52].

ATP and adenosine levels are controlled by a sophisticated cascade of cell surface-localized enzymes collectively known as ectonucleotidases. These enzymes hydrolyze nucleoside triphosphates, diphosphates, and monophosphates to their respective nucleosides. There are five major families of ectonucleotidases: ectonucleoside triphosphate diphosphohydrolases (E-NTPDases), ectonucleotide pyrophosphatase/phosphodiesterases (E-NPPs), alkaline phosphatases, ectonucleoside diphosphokinase (E-NDPK), and ecto-5′-nucleotidase. 

Ectonucleotidases are membrane-bound enzymes with catalytic site located in the extracellular medium. NTPDase1, 3 and 8 slightly prefer ATP over ADP by a ratio of 1, 3, and 2, respectively, whereas NTPDase 2 prefers triphosphonucleosides [53]. Ecto-5′-nucleotidase hydrolyses monophosphate nucleosides, for example, AMP, and is directly involved in adenosine production in the synaptic cleft. These enzymes promote the complete ATP hydrolysis to adenosine and may play an important role in physiological and pathological conditions, controlling the activation and the availability of ligands to nucleotide and nucleoside receptors.

Thus, the effect of the TH on the purinergic system can influence the actions mediated by the adenine nucleotides allowing us to understand the features involved in thyroid dysfunctions.

## 3. Thyroid Hormones and Purinergic System 

It has been previously shown that the sensibility to inhibitory agents, such as adenosine is increased in the hypothyroid status [54].

Considering that TH modulate a number of physiological functions in CNS, including development, function, expression of adenosine A1 receptors, and transport of neuromodulator adenosine, we investigated the effects of the hyperthyroidism and hypothyroidism on the hydrolysis of the ATP to adenosine in the synaptosomes of hippocampus and cerebral cortex of rats in different developmental phases [55, 56]. Hyperthyroidism was induced in male Wistar rats from different ages by daily injections of l-thyroxine (T4) for 14 days [55]. Hypothyroidism was induced in these rats by thyroidectomy and methimazole (0.05%) added to their drinking water for 14 days. Neonatal hypothyroidism was induced by adding 0.02% methimazole in the drinking water from day 9 of gestation and continually until 14 days old [56]. In these studies, it was possible to observe that hyperthyroidism decreased the hydrolysis of ATP to adenosine in both hippocampus and cerebral cortex synaptosomes, while hypothyroidism increased the AMP hydrolysis to adenosine in both hippocampus and cerebral cortex synaptosomes from rats at all aged tested. These results suggest that the increase of ATP availability as an excitatory neurotransmitter and the potential decrease of adenosine as an inhibitory molecule can be associated with some hyperthyroidism symptoms [55]. In contrast, the potential increase in adenosine levels in brain synaptosomes from hypothyroid rats may explain some inhibitory effects observed in hypothyroidism [56].

 Furthermore, the results observed in synaptosomes were also seen in hippocampal and cortical slices from hypothyroid and hyperthyroid rats [57]. In this study, the increase in the ATP, ADP, and AMP hydrolysis was reverted by T4 replacement in brain slices from hypothyroid rats. Moreover, hypothyroidism increased the expression of NTPDase1 and 5′-nucleotidase, whereas hyperthyroidism decreased the expression of this enzyme in hippocampus of adult rats. 

Thus, hyperthyroidism and hypothyroidism affect the extracellular nucleotides balance and adenosine production, possibly interfering in neurotransmitter release, development, and other physiological processes. 

**Figure 1:**
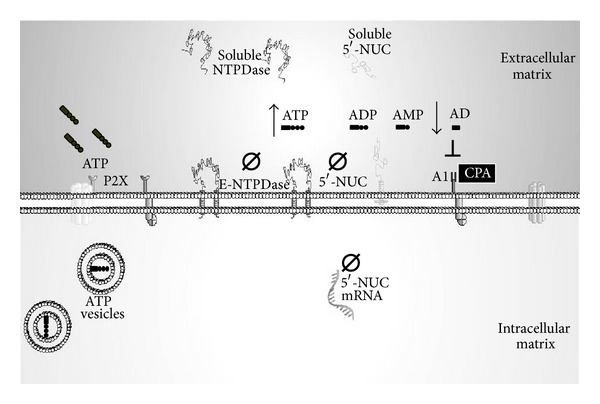
Schematic figure representing our data relating the purinergic system with hyperthyroidism in the CNS. The figure demonstrates that the induction of hyperthyroidism resulted in a significant reduction in the NTPDases and 5′-nucleotidase (5′-NUC) activities which degrade the ATP to adenosine in the brain rats chronically treated with T4 [55]. This result was also observed in 5′-nucleotidase expression in brain of hyperthyroid rats [57]. This leads to an increase of ATP availability as an excitatory neurotransmitter and the reduction of adenosine as an inhibitory neuromodulator in different brain fractions studied. The reduction in the extracellular adenosine levels may reduce the inhibitory effects mediated by adenosine A1 receptors. This relation was demonstrated in hyperthyroidism, once that N6-cyclopentyladenosine (CPA), a selective agonist of the A1 adenosine receptor, reverted the hyperalgesia induced by hyperthyroidism and increased the total antioxidant reactivity in hyperthyroid brain [58]. Moreover, the effects observed in the nervous system were also observed in blood serum of hyperthyroid rats, indicating a possible use of soluble enzyme markers for this thyroid disease [55, 59].

In Figure 1, we can observe a representation of the results describing the effects of hyperthyroidism on the purinergic system in the CNS.

It has been shown that ATP is involved in the interaction between neurons and glial cells, homeostasis, and inflammatory reactions in the brain [50]. In cultured astrocytes, ATP induces differentiation, proliferation, and reactive astrogliosis in injured tissue [51]. Therefore, the relation between the ectonucleotidases and TH was also investigated in brain cell culture from rats submitted to neonatal hypothyroidism and demonstrated that the adenine nucleotides hydrolysis was altered in cerebellar and hippocampal astrocytes from immature hypothyroid rats. Therefore, the imbalance in the ATP and adenosine levels in astrocytes during brain development may contribute to some of the effects described in neonatal hypothyroidism [60].

Moreover, the effects observed in the nervous system, were also observed in blood serum of rats at different developmental stages [55, 56, 59]. These results are important for the possible use of enzyme markers for these thyroid diseases.

There is increasing evidence that adenosine and ATP may act as pain neuromodulators in the spinal cord [61, 62]. Adenosine has antinociceptive properties in experimental and clinical situations [41, 63], while ATP exerts pronociceptive actions in different pain models [64]. Considering this relation, our research group investigated the hydrolysis of ATP to adenosine in synaptosomes from spinal cord in parallel with the nociceptive response of rats at different ages after hyperthyroidism and hypothyroidism induction [58, 65]. Hypothyroidism elicited a significant increase in AMP hydrolysis to adenosine in synaptosomes from spinal cord in parallel with a pronounced analgesic response. Thus, a possible increase in the adenosine levels could justify the analgesic response observed in hypothyroid rats [58]. Similarly, the increase in the nociceptive response in hyperthyroid rats could be justified by the decrease in the hydrolysis of ATP to adenosine in the spinal cord from these rats, emphasizing the relation between ectonucleotidases and nociception in thyroid diseases [65]. 

It is known that the protective and inhibitory effects of adenosine are mediated by A1 receptors. These inhibitory effects occur in such processes such as neurotransmitter release, nociception, motor activity, exploratory behavior, anxiety, and reactive oxygen species (ROS) effects. Therefore, in a previous study we investigated the consequences of the administration of a specific agonist of adenosine A1 receptor on behavioral and biochemical parameters after hyperthyroidism induction [58]. In this study, we used a selective adenosine A1 receptor agonist, N6-cyclopentyladenosine (CPA), in hyperthyroid rats. The results demonstrated that CPA reverted the hyperalgesia induced by hyperthyroidism and decreased the exploratory behavior, locomotion, and anxiety in hyperthyroid rats. Furthermore, CPA decreased lipid peroxidation in hippocampus and cerebral cortex of control rats and in cerebral cortex of hyperthyroid rats. CPA also increased the total antioxidant reactivity in hippocampus and cerebral cortex of control and hyperthyroid rats. These results suggest that some of the hyperthyroidism effects are subjected to regulation by adenosine A1 receptor, demonstrating the involvement of the adenosinergic system in this pathology.

The relation between the TH and the purinergic system was also demonstrated in testis. Since, the Sertoli cells present in the seminiferous tubules provide physical support to germ cells, form the blood testis barrier, and secrete protein products which are thought to be essential for the maintenance and control of spermatogenesis [66], the hormonal factors controlling the duration of Sertoli cell proliferation are critical determinants of fertility [67]. Alterations in thyroid activity are frequently associated with changes in male reproductive functions, since hypothyroidism, induced or occurring soon after birth, is associated with a marked delay in sexual maturation and development [68]. In addition to the hormonal modulation of Sertoli cell functions, there are several reports evidencing that extracellular adenine nucleotides can modulate responses through the purinoceptors present in these cells [69–73]. In a previous study, the enzymes NTPDase 1, 2, and 3 were detected by RT-PCR in Sertoli cell cultures [26]. However, hypothyroidism was not able to alter the expression of these enzymes, but significantly decreased the extracellular ATP and ADP hydrolysis. These findings demonstrate that TH modify NTPDase activities in hypothyroid Sertoli cells, probably via nongenomic mechanisms and, consequently, may influence the reproductive function throughout development [26]. 

Furthermore, the maintenance of the normal vascular function also has been related with the purinergic system [74]. TH has an inhibitory effect on the platelet aggregation in vitro [75] and in vivo [76]. Accordingly, hypothyroid patients present an increase in platelet aggregation, while hyperthyroid patients have a decrease in this process. Moreover, hypothyroidism has been associated with diseases such as artherosclerosis and thrombosis [77, 78]. Depending on the concentration, ATP can stimulate or inhibit platelet aggregation [79]. On the other hand, ADP is a potent platelet-recruiting factor inducing platelet aggregation [29]. We previously demonstrated a significant increase in the AMP hydrolysis in platelets from hyperthyroid rats and a decrease of the hydrolysis of this nucleotide in response to hypothyroidism. Besides, the T4 replacement significantly reversed the inhibition of the AMP hydrolysis observed in hypothyroid rats. These findings indicate that the thyroid disorders affect the 5′-nucleotidase activity and consequently can alter the adenosine levels in a reversible manner in platelet fraction. Since, adenosine is able to inhibit platelet aggregation and acts as a potent vasodilator, these results can contribute to a better comprehension of the vascular events described in thyroid disorders [79].

There was undertaking studies to evaluate the role of TH in the extracellular nucleotide hydrolysis by ecto-NTPDase in the cardiac cells. A previous study demonstrated that T3 increase the ATP and ADP hydrolysis and the NTPDase 3 messenger RNA levels in primary cultures of rat ventricular myocytes [80]. Since T3 promotes an increase in the contractile protein, leading to cardiac hypertrophy, it is tempting to postulate that the increase in ATP hydrolysis and the decrease in the extracellular levels signify an important factor for prevention of excessive contractility [80].

It was recently demonstrated that hypothyroidism significantly increases the 5′-nucleotidase activity in the soluble fraction of cardiac tissue in a reversible manner. These data indicates that TH can influence adenosine production and possibly contribute to the cardioprotective effect and the maintenance of cardiac function under TH privation [81].

In the cardiac tissue, the intra- and extracellular levels of adenosine can be regulated through hydrolytic deamination to inosine by adenosine deaminases (ADA) [82]. In a previous study, ADA inhibition significantly enhanced the efficiency of A1 adenosine receptor signaling pathway in the hyperthyroid guinea pig atrium. This result suggests that elevated intracellular adenosine level caused by ADA inhibition may improve the suppressed responsiveness to A1 adenosine receptor agonists associated with the hyperthyroid state [83]. 

Adenosine also decreases O_2_ demand by reducing the heart rate (a negative chronotropic effect), atrioventricular conduction (a negative dromotropic effect), and contractile force (a negative inotropic effect). It increases O_2_ supply by causing coronary vasodilatation [84]. Studies have also shown that the levels of A1 adenosine receptors in the heart correspond to heart rate and to cardiac efficiency [85]. 

Moreover, the ATP receptors, as well as P2X7 receptor, were also linked with other pathological situation such as thyroid cancer. The P2X7 receptor was upregulated in thyroid cancer lines. Thyroid cancer cells had at least a 3-fold higher intracellular ATP concentration and maintained at least a 3-fold higher extracellular ATP level, compared with control cells, suggesting that an enhanced P2X7 receptor function may be a feature of human thyroid cancer or can be used as a new potential marker of this disease [86]. Furthermore, the P2X7 receptor expression was also related to tumor size and capsular infiltration of papillary thyroid carcinoma, being described as possible predictors for lymph node metastasis in this kind of carcinoma [87]. 

Taken together, the studies mentioned in this text demonstrate the strong relationship between TH and the purinergic signaling in different physiological and pathological systems. Here, we mentioned the association between some symptoms of thyroid pathologies, with the enzymes responsible for the maintenance of ATP and adenosine levels, as well as with the A1 adenosine receptors. This relationship may, in some way, help us understand many of the effects observed in these pathologies.
